# Ether vs. Chloroform

**Published:** 1866-09

**Authors:** 


### ETHER VS. CHLOROFORM.

The use of chloroform as an anæsthetic was lately discussed in the Paris Academy of Science, in a paper by Dr. Petrequin, of Lyons. He favors the use of ether, and shows that the former inconveniences attending its use have been obviated. Dr. Petrequin says:

"These inconveniences were removed by the etherizing bag, invented by a physician of Lyons, and also by the improved methods devised for obtaining ether perfectly pure. At present the operation of etherizing proceeds in a most satisfactory manner. The patient lies in a horizontal posture, with his head slightly raised in order to prevent his swallowing any ether; about twenty-four grammes of ether are poured at once on the sponges of the bag; the patient is directed to inhale copiously; the orifice of the bag is then closed, and the dose of ether doubled. The patients eyes are carefully bandaged, and the profoundest silence is observed; in this way anæsthesia is promptly obtained without agitation. It is easy to prevent accidents by closely observing the circulation and breathing. In general the pulse is first accelerated, then becomes regular again; were it to become concentrated, irregular, or low, the inhalations should be stopped and air administered. As to the breathing, it is at first subdued, but soon becomes complete. If it were to become difficult or interrupted, the apparatus should be removed and the fan used. In this way there has not been a single death at Lyons by etherization for the last fourteen years, while, according to Dr. Velpeau's own showing, the use of chloroform is never unaccompanied by danger.

"To this paper Dr. Velpeau replied, that there had indeed been a few deaths by chloroform, but it was not quite certain that there had been none by ether. At all events, he (Dr. Velpeau) had for the last fifteen years been administering chloroform to several thousand patients, and had never seen any die from that cause."

